# Stop GnRH-agonist/GnRH-antagonist protocol: a different insight on ovarian stimulation for IVF

**DOI:** 10.1186/s12958-023-01069-7

**Published:** 2023-01-30

**Authors:** Raoul Orvieto

**Affiliations:** 1 Department of Obstetrics and Gynecology, Chaim Sheba Medical Center, Ramat Gan, Israel; 2 Sackler Faculty of Medicine, Tel-Aviv University, Tel Aviv-Yafo, Israel; 3 The Tarnesby-Tarnowski Chair for Family Planning and Fertility Regulation, Sackler Faculty of Medicine, Tel-Aviv University, Tel Aviv-Yafo, Israel

## Abstract

Ovarian stimulation (OS) is one of the key factors in the success of in vitro fertilization-embryo transfer (IVF-ET), by enabling the recruitment of numerous healthy fertilizable oocytes and, thereby, multiple embryos. The Stop GnRH-agonist/GnRH-antagonist (GnRH-ag/GnRH-ant), which offers all the advantages of using long suppressive GnRH-ag, with GnRH-ant, is in my opinion a valuable addition to the armamentarium of OS protocols. It allows cycle programming, better follicular synchronization and offers successful outcome in a variety of challenging cases such as poor responders, Poseidon group 4 poor prognosis patients, those with elevated peak progesterone (P) serum levels, poor embryo quality or repeated IVF failures.

## Background

Ovarian stimulation (OS) is one of the key factors in the success of in vitro fertilization-embryo transfer (IVF-ET), by enabling the recruitment of numerous healthy fertilizable oocytes and, thereby, multiple embryos. OS usually includes the co-administration of gonadotropins and gonadotropin-releasing hormone (GnRH) analogues; the two most commonly used protocols are the long GnRH-agonist (GnRH-ag), suppressive protocol and the multiple-dose GnRH-antagonist (GnRH-ant), OS protocol. When comparing these two OS protocols, the literature search yields conflicting results for live-birth rate [1]. A search for the optimal OS for poor responder (POR) patients is even more intricate and confusing. While many other OS strategies have been proposed for this frustrating situation, no compelling advantage for one stimulation protocol over another has been established [2].

### The stop GnRHAg/GnRh-ant protocol

**Fig. 1:**
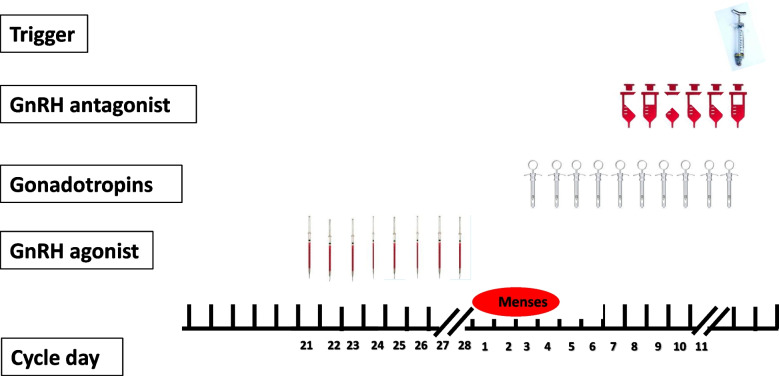
The Stop GnRH-agonist/GnRH-antagonist protocol

The stop GnRH-ag/GnRH-ant protocol (Fig. 1) is a valuable addition to the OS protocols armamentarium. The stop GnRH-ag/GnRH-ant protocol is comprised of daily GnRH-ag administration, starting in the midluteal phase, until the onset of menses and after confirmation of down-regulation by serum Estradiol (E2) levels and vaginal ultrasound measurements. Gonadotropins are initiated after two washout days, with tailored daily dose. GnRH-antagonist is added according to the individual program policy (fixed or flexible), and continued up to, and including, the day of triggering final follicular maturation. Final follicular maturation is triggered as soon as the majority of the leading preovulatory follicles have reached a diameter of 17 mm, preferably with a ratio of E2 level to number of leading preovulatory follicles of > 14 mm calculated to be lower than 100 pg/mL [3]. If a fresh transfer is planned, patients should receive luteal support according to the individual program policy.

The rationale behind the sequential treatment of the combined Stop GnRH-ag with multiple-dose GnRH-ant protocol stems from the advantages of its components. The long GnRH-ag protocol pretreatment results in better synchronized response and a scheduled cycle [4]. Moreover, since continuing the GnRH-ag during OS is often associated with a significant increase in the number of gonadotropin ampoules required for achieving adequate follicular development, its cessation might improve ovarian response and avoid the need of increasing the gonadotropin daily dose. GnRH-ag causes suppression of pituitary LH secretion for as long as 10 days after the last dose of the agonist [5], which, together with the immediate LH suppression provided by the GnRH-ant, will eliminate premature LH surge and may improve the quality of the embryos generated. Starting with rec FSH (with or without LH) preparation should result in a higher mature oocytes yield, as was already well demonstrated [6, 7]. An additional advantage is that final oocyte maturation may be triggered by GnRH-ag together with hCG (double/dual trigger), with potentially improved IVF outcome [8–10].

During routine clinical practice, physicians may encounter a variety of frustrating situations. In our practice, we started offering the Stop GnRH ag/GnRH-ant OS protocol to these patients, and compared their IVF outcome to their previous IVF attempt, thus, eliminating multiple bias factors.

### Patients with POR [11]

Thirty POR patients, defined according to the Bologna criteria, underwent a subsequent Stop GnRH-ago/GnRH-ant, within 3 months of the previous failed conventional IVF/ICSI cycle. Only “genuine” POR patients, defined as those who yielded up to 3 oocytes following OS with a minimal gonadotropin daily dose of 300 IU, were included. The Stop GnRH-ag/GnRH-ant OS protocol revealed significantly higher numbers of follicles > 13 mm on the day of hCG administration, higher numbers of oocytes retrieved, and top-quality embryos (TQE) with an acceptable clinical pregnancy rate (16.6%). Moreover, as expected, patients undergoing the Stop GnRH-agonist combined with multiple-dose.

GnRH-antagonist COH protocol required significantly higher doses and a longer duration of gonadotropins stimulation.

### Patients with elevated peak serum progesterone (P) levels [12]

Eleven patients with P elevation (> 3.1 nmol/L) during conventional IVF/intracytoplasmic sperm injection (ICSI), underwent a subsequent Stop GnRH-ag/GnRH-ant OS, within 3 months of the previous failed conventional IVF/ICSI cycle. Here again, the Stop GnRH-ag/GnRH-ant OS protocol revealed significantly lower peak progesterone levels, with significantly higher numbers of follicles > 13 mm in diameter on the day of hCG administration, oocytes retrieved, mature oocytes, and TQEs, with an acceptable clinical pregnancy rate (18.2%).

### Patients with repeated IVF failure and poor embryo quality [13]

Twenty-three patients with IVF failures and poor embryos quality during conventional GnRH-ant protocol, underwent a subsequent Stop GnRH-ag/GnRH-ant OS protocol. Following the stop GnRH-ag/GnRH-ant, while there were no in-between cycle differences in the number of oocytes retrieved or the number of MII oocytes, the Stop GnRH-ag/GnRH-ant OS protocol yielded significantly more TQE and a higher proportion of the number of TQE/number of MII oocytes retrieved. Seven clinical pregnancies (30.4%) were recorded.

Following the aforementioned experience, the Stop GnRH-ag/GnRH-ant has become one of the most prominent OS protocol in our practice, and was used with different modes of final follicular maturation trigger [8]. Since the GnRH-ag causes suppression of pituitary LH secretion for as long as 10 days after the last dose of the agonist [5], high responders might experience a suboptimal response to the GnRH-ag trigger. Prompted by the aforementioned information we decided to measure the LH level 12 h after the GnRH-ag/Dual trigger in patients undergoing the Stop GnRH-ag/GnRH-ant OS protocol. Five out of the 32 patients (15.6%) demonstrated suboptimal response as reflected by LH levels < 15 IU/L 12 h after GnRH-ag trigger. Moreover, those achieving a suboptimal response to the GnRH-agonist trigger (post-trigger LH < 15 mIU/mL) demonstrated significantly higher number of follicles and peak estradiol levels at the day of trigger, compared to those with optimal response (post-trigger LH > 15 mIU/mL). We therefore concluded that while the stop GnRH-ag/GnRH-ant protocol enables the substitution of hCG with GnRH-ag for final oocyte maturation, caution should be taken in high responders, where the dual trigger with small doses of hCG (1,000–1,500 IU) should be considered, aiming to avoid suboptimal response (post-trigger LH levels < 15 IU/L).

Finally, a recent proof of concept study [14] of Poseidon Group 4 [15] POR patients, offered the combined Stop GnRH-ag/ GnRH-ant and letrozole priming (for 5–7 days from confirmation of downregulation, until the start of OS), combining pre-treatment pituitary suppression and androgen-modulating agents. Patients achieved significantly higher number of follicles > 13 mm on the day of hCG administration and higher number of oocytes retrieved, with non-significantly more TQEs and a reasonable clinical pregnancy rate. Maintaining pituitary suppression after achieving down regulation, provides the “5–7 days pause,” for the letrozole priming. This pause allows the development of additional follicular wave while enabling letrozole to increase intrafollicular androgens levels [16, 17] and augment FSH receptor expression on granulosa cells with the consequent increase in the number of FSH-sensitive antral follicles [18–20].

The studies regarding the stop GnRH-ag/GnRH-ant protocol have been conducted in a single university affiliated tertiary medical center, by a professional and consistent team. The limitations of the aforementioned studies are the relatively small sample size, and the use of more medications which might be more expensive, and also it complexity might be more confusing for patients.

## Conclusions

The Stop GnRH ag/GnRH-ant is a valuable addition to the armamentarium of OS protocols. It offers all the advantages of using long suppressive GnRH-ag, with GnRH-antagonists. Moreover, it allows cycle programming, better follicular synchronization and may offer an improved outcome in a variety of challenging cases such as poor responders [11], Poseidon group 4 poor prognosis patients [14], those with elevated peak P serum levels [12], poor embryo quality or repeated IVF failures [13]. In addition, this protocol might still protect from severe ovarian hyperstimulation syndrome (OHSS) by maintaining the option to substitute hCG with GnRH-ag for final follicular maturation in patients at risk of OHSS [10]. Randomized controlled studies to validate the effectiveness of the Stop GnRH-ag/ GnRH-ant as compared to the long GnRH-agonist or multiple-dose GnRH-antagonist OS protocols, are needed to strengthen this concept.

## Data Availability

N/A.
